# Structural and Functional Alterations of Gut Microbiota in Males With Hyperuricemia and High Levels of Liver Enzymes

**DOI:** 10.3389/fmed.2021.779994

**Published:** 2021-11-19

**Authors:** Shifeng Sheng, Jingfeng Chen, Yuheng Zhang, Qian Qin, Weikang Li, Su Yan, Youxiang Wang, Tiantian Li, Xinxin Gao, Lin Tang, Ang Li, Suying Ding

**Affiliations:** ^1^Health Management Center, The First Affiliated Hospital of Zhengzhou University, Zhengzhou, China; ^2^College of Public Health, Zhengzhou University, Zhengzhou, China; ^3^Department of Nephropathy, The First Affiliated Hospital of Zhengzhou University, Zhengzhou, China; ^4^Gene Hospital of Henan Province, The First Affiliated Hospital of Zhengzhou University, Zhengzhou, China

**Keywords:** uric acid, liver enzymes, short-chain fatty acids, purine, aromatic amino acids, gut microbiota, equol

## Abstract

**Objective:** To investigate the correlation between the structure and function alterations of gut microbiota and biochemical indicators in males with hyperuricemia (HUA) and high levels of liver enzymes, in order to provide new evidences and therapeutic targets for the clinical diagnosis and treatment of HUA.

**Methods:** A total of 69 patients with HUA (HUA group) and 118 healthy controls were enrolled in this study. Their age, height, waist circumference, weight, and pressure were measured. The clinical parameters such as fasting plasma glucose (FBG), aspartate aminotransferase (AST), alanine aminotransferase (ALT), serum uric acid (SUA), serum creatinine (Scr), total cholesterol (TC), triglyceride (TG), low-density lipoprotein (LDL), high-density lipoprotein (HDL), white blood cell (WBC), platelet (PLT), and absolute value of neutrophils (NEUT) were examined. We used whole-genome shotgun sequencing technology and HUMAnN2 MetaCyc pathway database to detect the composition and pathways of the gut microbiota. The main statistical methods were student's *t* test, chi-square tests, and Wilcoxon rank sum test. The correlations among bacterial diversity, microbial pathways, and biochemical indicators were evaluated by the R function “cor.test” with spearman method.

**Results:** The gut bacterial diversity in HUA group reduced significantly and the community of the microbiota was of significant difference between the two groups. The pathways that can produce 5-aminoimidazole ribonucleotide (PWY-6122, PWY-6277, and PWY-6121), aromatic amino acids, and chorismate (COMPLETE-ARO-PWY, ARO-PWY, and PWY-6163) were enriched in the HUA group; while the pathways that can produce short-chain fatty acids (SCFAs, such as CENTFERM-PWY and PWY-6590) and the gut microbiotas that can produce SCFAs (*Roseburia hominis, Odoribacter splanchnicus, Ruminococcus callidus, Lachnospiraceae bacterium 3_1_46FAA, Bacteroides uniformis, Butyricimonas synergistica*) and equol (*Adlercreutzia equolifaciens*) were enriched in healthy controls.

**Conclusion:** The structure and function of the gut microbiota in males with HUA and high levels of liver enzymes have altered apparently. In-depth study of related mechanisms may provide new ideas for the treatment of HUA.

## Introduction

Hyperuricemia (HUA) is a group of heterogeneous chronic metabolic pathological conditions caused by purine metabolism disorder and/or uric acid excretion disorder. The excessive production and/or decreased excretion of uric acid level caused by any cause can lead to the occurrence of HUA. HUA is not only the direct cause of gout, but closely related to the onset and progression of many diseases such as diabetes, metabolic syndrome, chronic kidney disease, cerebrovascular-related diseases, and hyperlipidemia. Studies have confirmed that uric acid level is an independent risk factor leading to the progression of cardiovascular-related diseases such as hypertension and coronary heart disease, and it has an important impact on its prognosis (1). The latest research data show that the prevalence of HUA in the Chinese population has risen from less than 1.5% in the early 1980s to as high as 13% (2), and it is still rising and the incidence is showing a younger trend. Therefore, it is of great significance to study in depth the pathogenesis and further explore effective treatment measures of HUA.

Healthy people excrete uric acid in two ways mainly, of which 70% is excreted through the kidney, and the remaining 30% is excreted through the intestine (3). Intestinal microbes are a group of microorganisms designated to be planted in the intestinal tract, which have a symbiotic relationship with the human host and have a variety of important physiological functions such as participation in digestion, metabolism, promotion of nutrient absorption, synthesis of trace elements, and regulation of immune function. They are of great significance for the maintenance of our health (4). Researches in recent years have shown that changes in intestinal microbiota are not only related to diabetes, obesity, hypertension, and other diseases, but closely related to the occurrence of HUA. Studies have shown that changes in the gut microbiota and HUA promote each other and jointly aggravate the disease progression. For example, the intestinal microbiota can participate not only in the catabolism of purine and uric acid through the regulation of *Escherichia coli* and *Lactobacillus* (5–7), but also in the other two ways to affect the excretion of uric acid such as the production of short-chain fatty acids (SCFAs) (8–10) and changes in the number and distribution of uric acid transporters (11, 12) in the body. Besides, purine metabolism increases the production of uric acid, which can induce intestinal oxidative stress and then further promotes the infiltration of inflammatory cells and causes chronic inflammation in the intestinal tract. However, there are not many studies on the gut microbiota as an entry point to investigate HUA, and most of them are animal experiments. In view of the fact that most patients with HUA are asymptomatic and the intestinal microecology can change correspondingly in other disease states, it is more important and suitable to select the asymptomatic people who take physical examination as the research subjects to explore the correlation between HUA and gut microbiota. This study intends to explore the relationship among the serum uric acid (SUA) level, liver enzymes, and the detection of intestinal microbes in the physical examination population, and to provide more evidence support for finding the relationship among the serum uric acid, liver enzymes, and intestinal microecology.

## Materials and Methods

### Study Design

**Figure 1:**
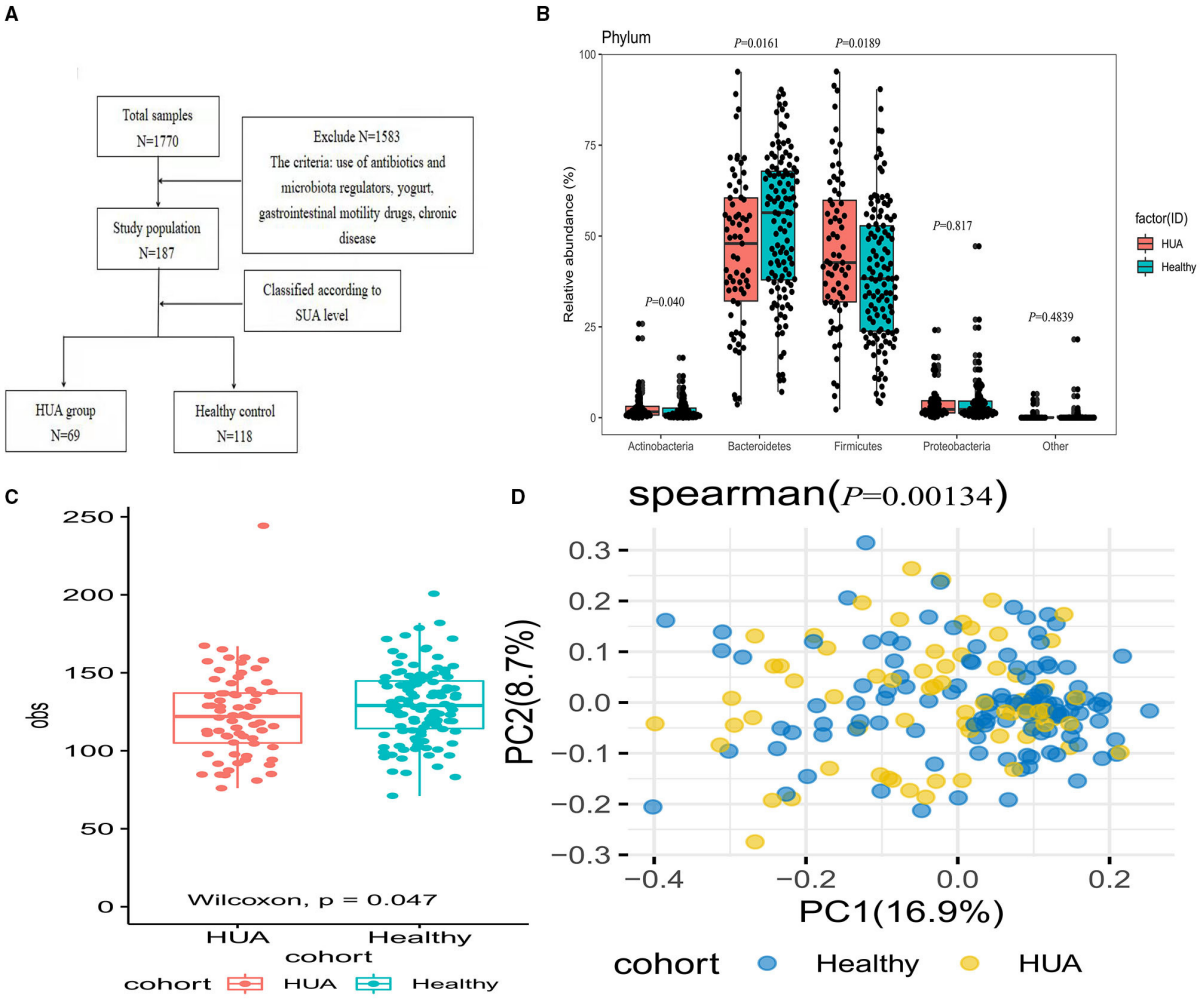
Comparison of the microbial community between HUA group and healthy controls. **(A)** Enrollment flow; **(B)** The abundance of gut microbiota between HUA and healthy controls at the phylum level; **(C)** Alpha diversity was analyzed at the species level by obs index between HUA group (*N* = 69) and healthy controls (*N* = 118); **(D)** Beta diversity by Pearson distance between the two groups.

Totally, 89 persons and 118 participants were randomly enrolled in HUA group and healthy controls from the adult population who underwent the physical examination in the Department of health management center, the First Affiliated Hospital of Zhengzhou University, namely HUA group and healthy control group (healthy controls). According to the multidisciplinary expert consensus on the diagnosis and treatment of HUA-related diseases in China, the diagnostic criteria of HUA was defined as the fasting plasma uric acid value >420 μmol/L(7 mg/dL) on different days, excluding concomitant diabetes, tumors and hematological diseases. Exclusion criteria: persons with clear gastrointestinal diseases or severe diseases of cardiovascular, respiratory, kidney, and other systems; pregnant and lactating woman; minors under 18 years old; antibiotics and microbiota regulators, yogurt, gastrointestinal motility drugs, and other preparations that may affect the intestinal microbiota had been used in the past 2 months. According to the test, 89 people had HUA, including 2 females, 11 persons used antibiotics and yogurt preparations recently, and 7 people were of high blood sugar or used hypoglycemic drugs or antihypertensive drugs. After excluding the above population, 69 people were enrolled in HUA group (Figure 1A). The research protocol was approved by the Ethics Committee of the First Affiliated Hospital of Zhengzhou University (2018-KY-56 and 2018-KY-90), and all of the study subjects signed an informed consent form.

### General Information and Related Biochemical Indicators

The height, weight, waist circumference (Computerized body scale, SK-X80), and blood pressure (OMRON Medical automatic electronic blood pressure monitor, HBP-9021) were measured by designated staff after all subjects are enrolled in the group. Each indicator was measured for three times and the average value was taken, and then the body mass index (BMI) is calculated according to the following formula: BMI = weight Kg/ (height m)^2^. Every study subject was required to fast and no water for 8–12 h and got 5 mL of cubital venous blood on an empty stomach in the morning. All the test tubes were placed in the specimen box and sent to the clinical central laboratory within 20 min by the logistics trolley transported by the hospital's special specimens after the blood was drawn. UniCel DxI 800 Immunoassay System from Beckman Coulter was used to test the blood samples. The following laboratory tests were collected such as fasting plasma glucose (FPG), aspartate aminotransferase (AST), alanine aminotransferase (ALT), glutamyl transpeptidase (GGT), total bilirubin (TBIL), indirect bilirubin (IBIL), SUA, serum creatinine (Scr), total cholesterol (TC), triglyceride (TG), low-density lipoprotein (LDL), high-density lipoprotein (HDL), white blood cell (WBC), platelet (PLT), and absolute value of neutrophils (NEUT).

### Collection of Stool Samples

Approximately 1 g of fresh stool specimen was collected from each study subject, placed the microbial sample preservation tube at −20°C, and transferred to −80°C for cryopreservation within 30 min. All blood and stool samples were collected before 10 o'clock on the day.

### Statistical Analysis

R program (version 4.0.5) was used for the statistical analyses. Before analyzing the differences of the gut microbiota, we removed species with low expression levels and occurrence rates (positivity rates <10%). The laboratory test, demography, bacterial species, and pathways were analyzed by standardized statistical test methods. The main statistical methods were student's *t* test, chi-square tests, and Wilcoxon rank sum test. The continuous variables were expressed as mean ± SD ($\bar{x}\pm$ s) and classified variables were represented by counts. Normal test and homogeneity test were applied to analyze the difference between groups, and *P* ≥ 0.05 was selected as the normal and homogeneity variances. Then, we used parametric test (*t* test) or non-parametric test (rank sum test) and *P* < 0.05 was regarded as statistically significant. The correlations among bacterial diversity, microbial pathways, and biochemical indicators were evaluated by the R function “cor.test” with Spearman method. The “ADE4” package in R program was applied to perform the principal coordinate analysis (PCoA) and the “vegan” package was used to calculate observed species number (obs) and Spearman index for each sample.

### DNA Extraction, Shotgun Metagenomic Sequencing, and Reads Quantity Control

We extracted DNA from 1770 stool samples according to the operating instructions of the MagPure Stool DNA KF kit. All samples were tested using DNA nanospheres (DNB) based on DNA libraries construction and probe-anchored synthesis technology (cPAS) (MGI2000, MGI, Shenzhen, China) based on 100 bp paired-end reads of shotgun metagenomic sequencing. The overall accuracy (OA ≥ 0.8) control strategy were used to make sure the quality control (QC) of raw sequencing reads to filter out low-quality reads (13, 14). SOAPaligner/soap2 was applied to filter out human reads and hg19 was selected as the standard point of the high-quality reads (identity ≥ 0.9) (RRID:SCR_005503).

### Microbiota Composition and Function Profiling

The taxonomic annotation and quantification on the base of MetaPhlAn2 with default settings were performed (15), and the generating gut microbial profiling of all levels such as bacteria, archaea, viruses, and eukaryotes were included. The HMP Unified Metabolic Analysis Network 2 (HUMAnN2) and National Center for Biotechnology Information (NCBI) (nlm.nih.gov) database (2014 Edition) were applied to annotate the nonredundant gene set and the functional genes into Kyoto Encyclopedia of Genes and Genomes (KEGG) metabolic pathway, and then the corresponding metabolic pathways were generated (13, 14).

## Results

### Clinical Characteristics of Subjects

A total of 69 patients with HUA (HUA group) and 118 healthy participants (healthy controls) were included in this cross-sectional cohort study. The level of SUA in the HUA group was significantly higher than that of the control group, accompanied by WC, BMI, AST, ALT, GGT, Scr, and TG were all higher than the control group, while HDL was lower than the control group. The differences were statistically significant (all *P* < 0.05) (Table 1).

**Table 1:** The major demographic and serum features in males of HUA group and healthy controls.

**Feature**	**HUA (*n =* 69)**	**Healthy (*n =* 118)**	***P***
Age (year)	41.217 ± 10.345	43.805 ± 11.144	0.111
WC (cm)	92.622 ± 7.320	87.451 ± 8.352	<0.001***
BMI (Kg/m^2^)	26.951 ± 2.880	24.595 ± 3.120	<0.001***
FPG (mmol/L)	5.194 ± 0.535	5.092 ± 0.464	0.190
DBP (mmHg)	81.319 ± 9.118	78.715 ± 9.929	0.070
SBP (mmHg)	129.493 ± 13.142	126.963 ± 14.468	0.223
AST (U/L)	24.841 ± 10.084	22.102 ± 5.641	0.041*
ALT (U/L)	33.942 ± 24.315	23.949 ± 9.718	0.002**
GGT (U/L)	47.565 ± 37.338	29.415 ± 23.757	<0.001***
TBIL (μmol/L)	13.126 ± 7.503	13.331 ± 7.422	0.857
IBIL (μmol/L)	8.03 ± 5.303	8.153 ± 5.699	0.883
SUA (μmol/L)	470.652 ± 48.647	330.212 ± 50.939	<0.001***
Scr (μmol/L)	80.681 ± 10.830	75.297 ± 9.111	<0.001***
TC (mmol/L)	4.935 ± 0.876	4.724 ± 0.902	0.119
TG (mmol/L)	2.311 ± 1.431	1.381 ± 0.756	<0.001***
LDL (mmol/L)	3.056 ± 0.778	2.94 ± 0.790	0.329
HDL (mmol/L)	1.204 ± 0.227	1.408 ± 0.346	<0.001***
WBC (10^9^/L)	6.443 ± 1.267	6.051 ± 1.406	0.051
PLT (10^9^/L)	230.899 ± 49.458	221.398 ± 48.270	0.203
NEUT (10^9^/L)	3.699 ± 1.089	3.477 ± 1.023	0.169
NLR	1.847 ± 0.722	1.829 ± 0.619	0.858

WC, waist circumference; BMI, body mass index; FPG, fasting plasma glucose; DBP, diastolic blood pressure; SBP, systolic blood pressure; AST, aspartate aminotransferase; ALT, alanine aminotransferase; GGT, glutamyl transpeptidase; TBIL, total bilirubin; IBIL, indirect bilirubin; SUA, serum uric acid; Scr, serum creatinine; TC, total cholesterol; TG, triglyceride; LDL, low-density lipoprotein; HDL, high-density lipoprotein; WBC, white blood cell; PLT, platelet; NEUT, absolute value of neutrophil; NLR, Neutrophil to lymphocyte ratio. ^*^, ^**^, and ^***^ denote P < 0.05, 0.01, and 0.001 with statistical significance compared with healthy controls, respectively.

### Community Diversity in HUA Group and Healthy Controls

As shown in Figure 1B, four main abundant phylas dominated in the gut microbiota were discovered at the phylum level and there were significant differences in the phylum of *Bacteroidetes, Firmicutes*, and *Actinobacteria* between the two groups (Supplementary Table 1). Alpha and beta diversity was used for the evaluation of the community diversity at the species level. In Figure 1C, alpha diversity by obs was significantly different between HUA group and healthy controls (Wilcoxon rank sum test; *P* < 0.05). We then investigated the community variance among participants based on beta diversity by Pearson distance. Comparisons of the Pearson distance between the two groups showed significant differences in the first and second principal components (Wilcoxon rank sum test; *P* < 0.05) (Figure 1D).

### Analysis of the Composition of Gut Microbiota Between HUA and Healthy Controls and the Correlation Among the Gut Microbiomes With a Panel of Clinical Characteristics

#### Analysis of the Composition of Gut Microbiota Between HUA and Healthy Controls

**Figure 2:**
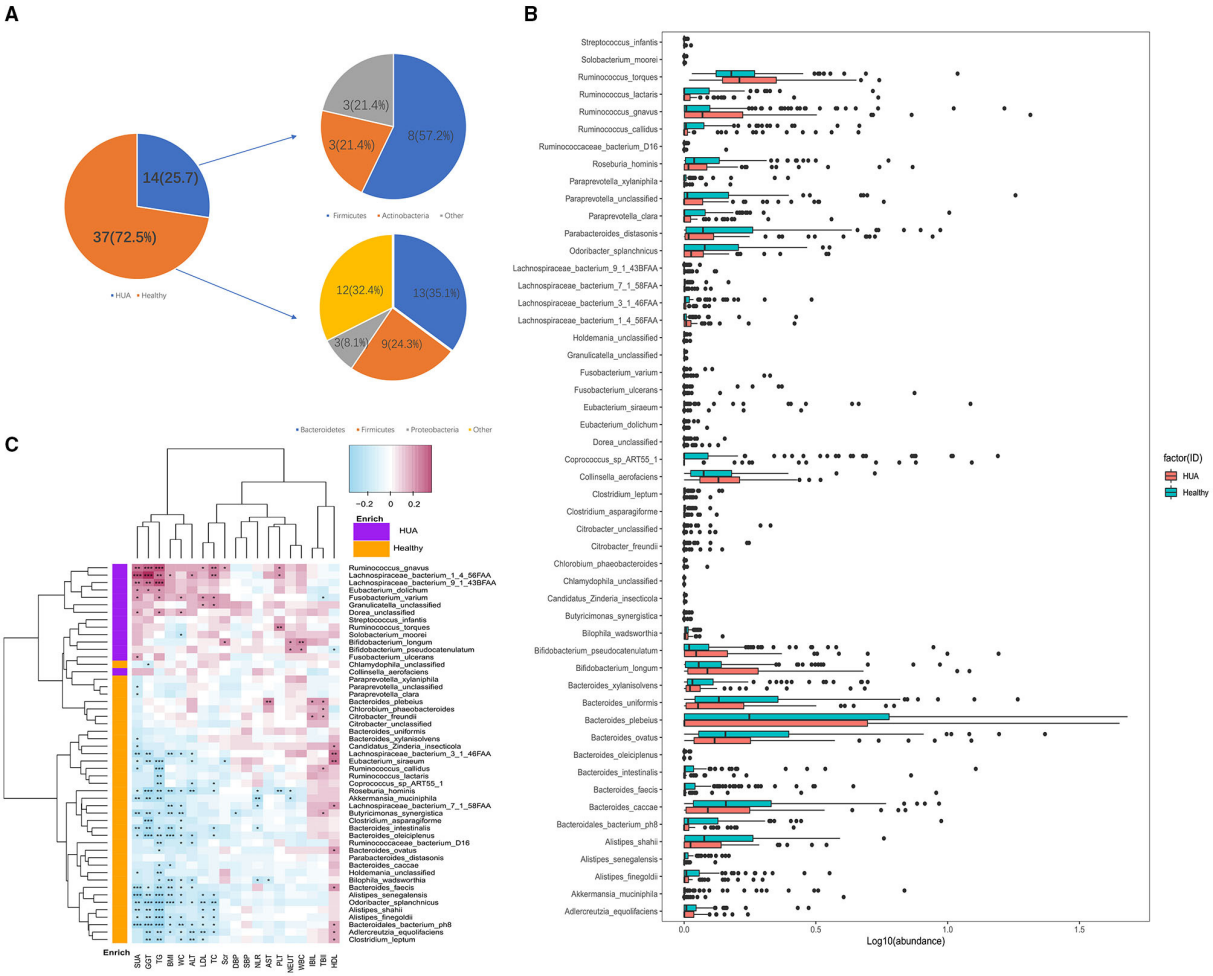
Microbiome differences between HUA group and healthy controls and the correlations with clinical characteristics. **(A,B)** Abundance of bacterial species that were of significant difference between HUA group and healthy controls. **(C)** Correlation matrix for bacterial species and clinical characteristics. Cells in red represented positive correlations while blue indicated negative. Cells with *, **, or *** asterisk represented *P* < 0.05, 0.01, or 0.001 respectively.

Totally 75 different species were identified between HUA group and healthy controls at the species level (Wilcoxon rank sum test; *P* < 0.05) (Supplementary Table 2), and 51 species were of significant difference after removing the low occurrence rate and low abundance species (*P* < 0.05; Figure 2A). In Figure 2A, we can see 37 species were enriched in the healthy controls and 14 species were enriched in the HUA group. From the view of species-level analysis, three high-abundance species (i.e., *Ruminococcus gnavus* and *torques* and *Lachnospiraceae bacterium 1_4_56FAA*) belonging to the *Firmicutes* were enriched in HUA group; and 12 species (i.e., *Alistipes finegoldii, senegalensis* and *shahii; Bacteroides caccae, faecis, intestinalis, ovatus, plebeius, uniformis* and *xylanisolvens, Odoribacter splanchnicus* and *Parabacteroides distasonis*) belonging to the *Bacteroidetes*; *five* species (i.e., Roseburia hominis, *Ruminococcus callidus* and *lactaris, Lachnospiraceae bacterium 7_1_58FAA*, and *Coprococcus_sp_ART55_1*) belonging to the *Firmicutes*; *three* species (i.e., *Adlercreutzia equolifaciens; Butyricimonas synergistica*, and *Akkermansia muciniphila*) belonging to other were enriched in healthy controls (Figure 2B).

#### Correlation Among the Gut Microbiomes With a Panel of Clinical Characteristics

Spearman's correlation analysis was applied to explore the association between the species abundances and clinical characteristics in this article. Totally 187 samples from HUA subjects and healthy controls were analyzed and 187 associations with original *P* values <0.05 were found out. In Figure 2C, we could see that except HDL, TBIL, and IBIL, most of the gut microbiomes enriched in HUA group had significant positive correlations with the clinical characteristics, while those microbiomes enriched in the healthy controls were highly negatively correlated with these clinical indexes (Supplementary Table 3). Among the clinical parameters, SUA and TG had the largest number of correlations with bacterial species (*n* = 26 and *n* = 28 respectively, *P* < 0.05), followed by GGT (*n* = 21, *P* < 0.05), WC (*n* = 19, *P* < 0.05), BMI (*n* = 15, *P* < 0.05), TC (*n* = 14, *P* < 0.05), ALT (*n* = 13, *P* < 0.05), LDL, and HDL (both *n* = 10, *P* < 0.05). Besides, *Roseburia hominis* had the most frequent correlations with clinical parameters among the 51 bacterial species (*n* = 10, *P* < 0.05), followed by *Odoribacter splanchnicus* and *Adlerreutzia equolifaciens* (both *n* = 8, *P* < 0.05), *Butyricimonas synergistica, Bacteroides intestinalis, Bacteroides faecis, Alistipes senegalensis, Alistipes fonegoldii, Lachnospiraceae bacterium 1_4_56FAA*, and *Ruminococcus gnavus* (all *n* = 7, *P* < 0.05), *Lachnospiraceae bacterium 3_1_46FAA* (*n* = 6, *P* < 0.05), among the above bacterial species, two were enriched in HUA group (*Ruminococcus gnavus* and *Lachnospiraceae bacterium 1_4_56FAA*).

### Functional Changes Brought About by the Microbiome in HUA Group and Healthy Controls

#### The Functional Differences From the Comparison of Different Participants

**Figure 3:**
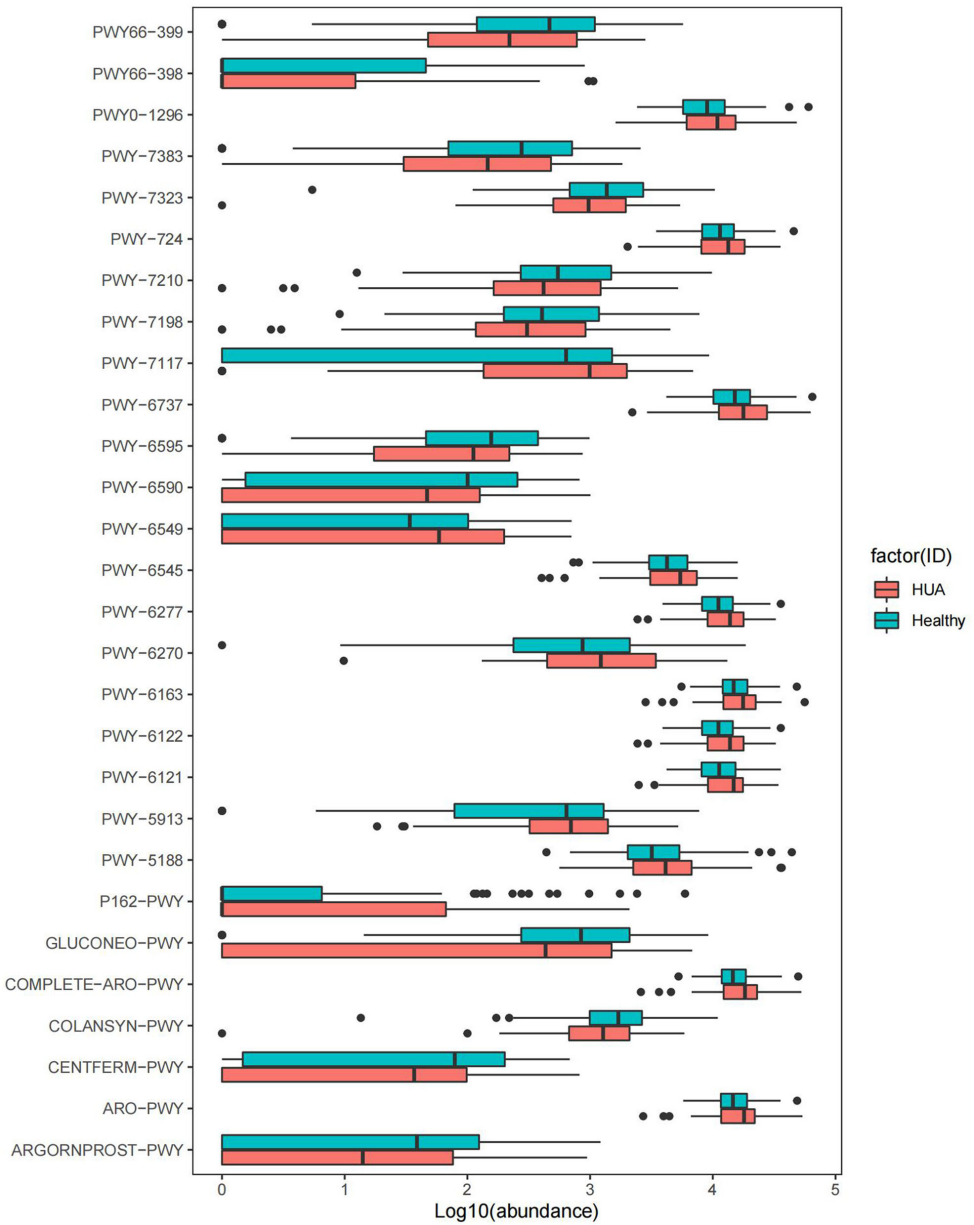
MetaCyc pathways with significant difference abundance between HUA group and healthy controls (Wilcoxon rank sum test, *P* < 0.05).

For each sample, MetaCyc pathway database by HUMAnN2 was further used to build the functional profiles with 494 pathways. Totally 41 pathways were identified in the comparison of MetaCyc pathway abundance between HUA group and healthy controls (Wilcoxon rank sum test; *P* < 0.05), and 28 pathways were of significant difference after removing the ones that were in low abundance and occurrence (Supplementary Table 4, Figure 3). Among the 28 pathways, 12 were enriched in healthy controls and 16 were enriched in HUA group. Within the 12 pathways in healthy controls, two were involved in the way of fermentation to SCFAs (CENTFERM-PWY and PWY-6590);one was related to the biosynthesis of GDP mannose (PWY-7323); five were related to the biosynthesis of gluconeogenesis (GLUCONEO-PWY and PWY66-399), pyrimidine deoxyribonucleotides (pwy-7198 and pwy-7210), and carbohydrate such as colanic acid (COLANSYN-PWY); two were responsible for energy generation (pwy66-398 tricarboxylic acid cycle, TCA) and energy metabolism (PWY-7383); one was related to superpathway of guanosine nucleotides degradation (PWY-6595); one was for the interconversion of arginine, ornithine, and proline (ARGORNPROST-PWY). Within the 16 HUA-enriched pathways, nine were related to the biosynthesis of 5-aminoimidazole ribonucleotide (PWY-6122, PWY-6277, and PWY-6121); pyrimidine deoxyribonucleotides (PWY-6545), aromatic amino acids, and chorismate (COMPLETE-ARO-PWY, ARO-PWY and PWY-6163) and tetrapyrrole (PWY-5188); one was responsible for L-glutamine biosynthesisIII, which is the way of nitrogen remobilization (PWY-6549); one was for purine ribonucleosides degradation (PWY0-1296); one was related to carbohydrate degradation, utilization, and assimilation (PWY-6737); one was involved in L-glutamate degradation (P162-PWY); one was for energy generation (PWY-5913); one was for the biosynthesis superpathway of L-lysine, L-threonine, and L-methionine II (PWY-724); and one was related to C4 photosynthetic carbon assimilation cycle (PWY-7117).

#### Correlation Between Microbial Pathways and a Panel of Clinical Characteristics

**Figure 4:**
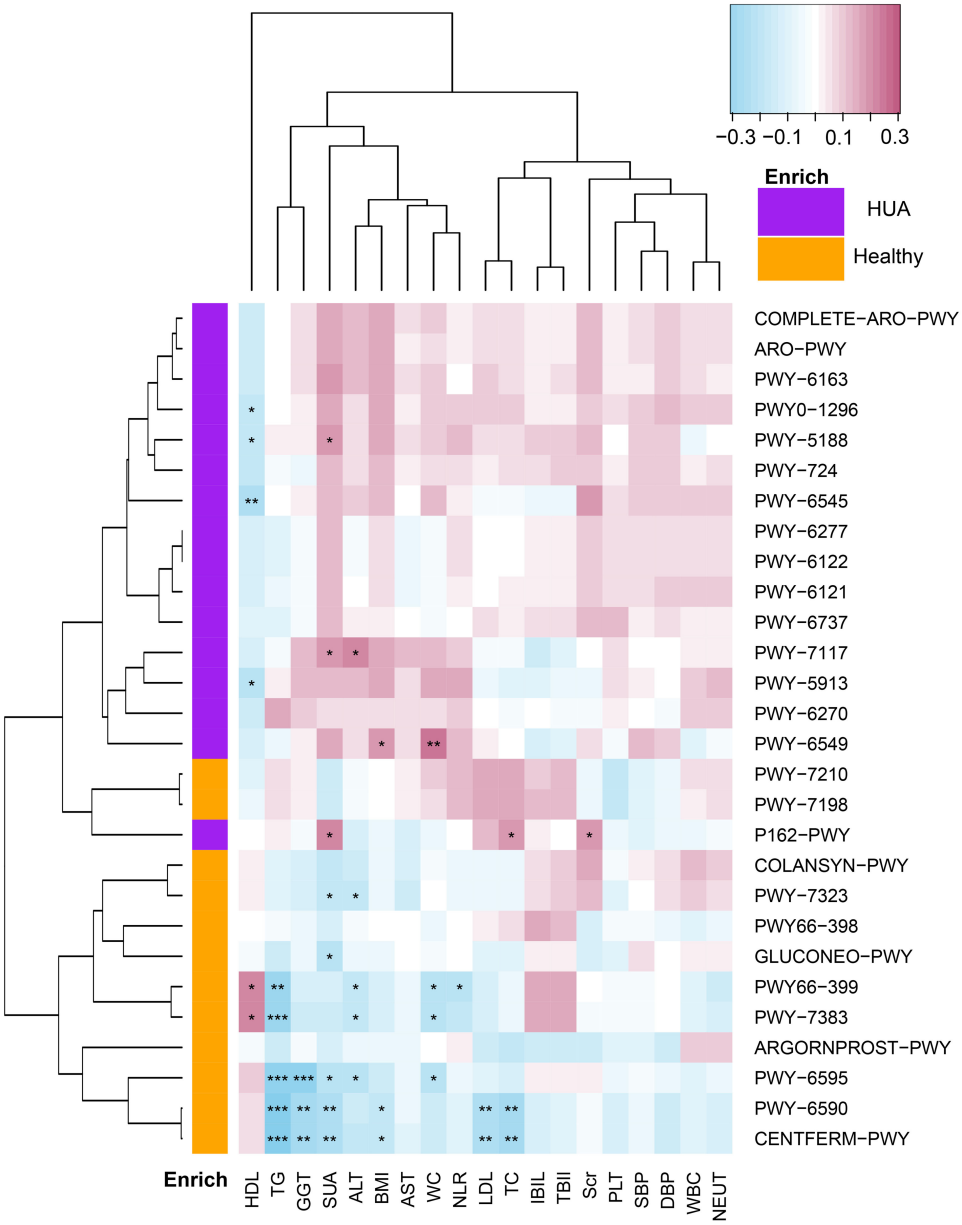
Spearman's correlation matrix for HUA group and healthy controls correlated pathways and clinical characteristics. Different cell color represented for relative correlation type (purple: negative, red: positive). Cells with *, **, or *** asterisk represented *P* < 0.05, 0.01, or 0.001 respectively.

Spearman's correlation analyses were applied for the analysis of MetaCyc pathways with significant difference and a panel of clinical characteristics, and then heatmaps were constructed (*P* < 0.05) (Figure 4, Supplementary Table 5). In Figure 4, we could see that except HDL, most of the pathways enriched in HUA group had highly positive correlations with the clinical characteristics, while those pathways enriched in the healthy controls mostly had significant negative correlations with these clinical indexes. Especially, SUA, TC, TG, LDL, BMI, and GGT had highly negative correlations with the pathway of producing SCFAs, such as PWY-6590 and CENTFERM-PWY (*P* < 0.05). Most of the above clinical index also had significantly negative correlation with PWY-6595, which is responsible for the degradation from purine to allantoin (*P* < 0.05). Besides, TG, ALT, and WC had highly negative correlations with the pathway of PWY66-399 and PWY-7383, while HDL is just the opposite (*P* < 0.05).

#### Correlation Between Microbial Pathways and a Panel of Bacterial Species

**Figure 5:**
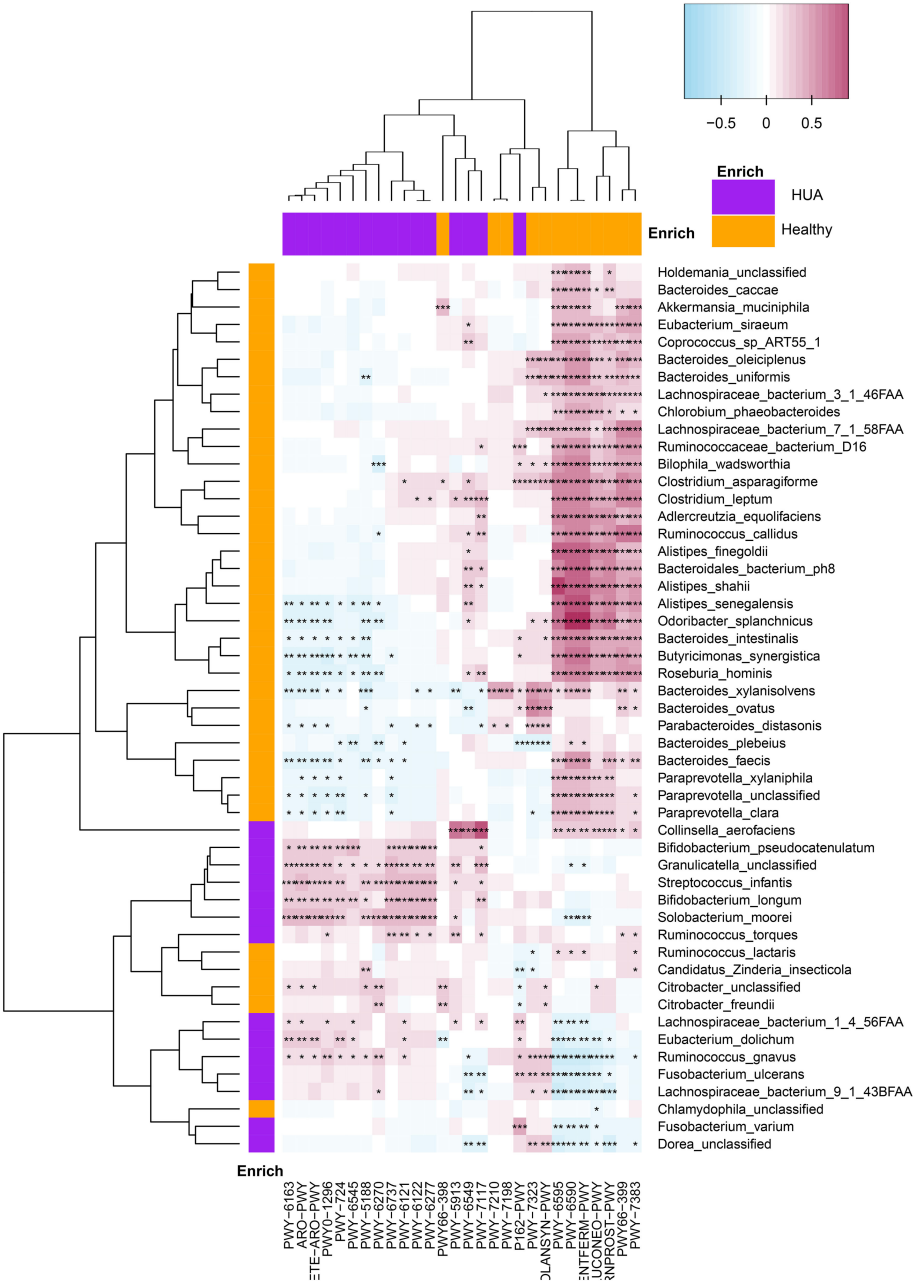
Spearman's correlation matrix for microbial pathways and bacterial species in HUA group and healthy controls. Different cell color represented for relative correlation type (purple: negative, red: positive). Cells with *, **, or *** asterisk represented *P* < 0.05, 0.01, or 0.001 respectively.

Spearman's correlation analyses were applied to analyze the correlations between bacterial species and microbial pathways (*P* < 0.05) and heatmaps were constructed with Spearman's correlations (Figure 5, Supplementary Table 6). Apparently, most of the bacterial species enriched in healthy controls had highly positive correlations with the pathways enriched in healthy group, and negative correlations with the pathways enriched in HUA group, while those pathways enriched in the HUA group had the opposite correlation trend. In particular, pwy-6590 and TFERM-pwy pathways had the stronger correlation with most of the bacterial species that can produce butyric acid enriched in healthy controls such as *Odoribacter splanchnicus, Roseburia hominis, Bacteroides uniformis, Lachnospiraceae bacterium 3_1_46FAA, Ruminococcus callidus*, and *Butyricimonas synergistica*. Besides, Lachnospiraceae bacterium 1_4_56FAA, *Ruminococcus gnavuswas and torques, Streptococcus infantis*, and *Solobacterium moorei* had significantly positive correlation with the pathway of producing 5-aminoimidazole ribonucleotide (pwy-6121, pwy-6122 and pwy-6277) and aromatic amino acids (pwy-6316, ARO-PWY and COMPLETE-ARO-PWY), and most of them are conditional pathogens.

## Discussions

It has been recognized that disturbances of the normal gut microbiome participate in the pathogenesis and progression of diverse chronic diseases, such as obesity (16), diabetes (17), hypertension (18), and liver cirrhosis (19), and the effect of gut microbiota in HUA has been explored gradually in recent years. For example, the current known interactions between the gut microbiota and HUA include that certain microbiota can regulate the synthesis and catabolism of purine and uric acid (such as *Escherichia coli* and *Lactobacillus*), produce some SCFAs or change the number and distribution of uric acid transporter. All of those studies have shown a strong correlation between the gut microbiota and HUA. However, most of them are animal experiments and the microbiota sequencing method is 16S rRNA, specific alterations of the gut microbiota composition and function between HUA (especially in males with HUA and high levels of liver enzymes) in asymptomatic people for physical examination have been rarely reported.

In view of the fact that most patients with HUA are asymptomatic and the intestinal microecology can change correspondingly in other disease states, our study is the first to select asymptomatic people for physical examination as the research subjects to explore the gut microbiome disturbances in males with HUA and high levels of liver enzymes and choose whole-genome shotgun sequencing technology, which are the two highlights and advantages of this research. Our results elucidated that there may do exist some targeted biomarkers of the gut microbiota and have the potential to be used as non-invasive, safe, and easy diagnostic tools for HUA. The new targeted biomarkers could be applied as a supplement to the traditional HUA diagnostic method to some extent.

In this study, we found that the level of SUA accompanied by WC, BMI, AST, ALT, GGT, Scr, and TG in the HUA group were significantly higher than that of the control group, while HDL was lower than the control group. Besides, the levels of FPG, blood pressure, TC, LDL, and inflammation indicators (WBC, PLT, NEUT, and NLR) were slightly, but not significantly, higher in the HUA group than in the healthy controls, TBIL and IBIL were of the opposite trend. Previous studies have confirmed that HUA is closely related to the occurrence of gout, metabolic syndrome, type 2 diabetes, hypertension, cardiovascular disease, chronic kidney disease, and so on, and it is an independent risk factor for the disease (20), which is consistent with our results. Numerous studies have shown that HUA is closely related to metabolic syndrome (obesity, hypertension, hyperlipidemia, insulin resistance), and the two factors are mutually causal and form a vicious circle (21–24). This comorbidity of chronic diseases suggests that there is a certain correlation between chronic diseases. Our results also suggest that HUA is associated with obesity and metabolism-related indicators, such as high levels of liver enzymes (5, 25), worse homeostasis in lipid and bilirubin metabolism, and poorer kidney function. Although the mean hepatic function was within the “current consensus normal reference range,” it had been already at a high level. Study has verified that when ALT exceeds 26 U/L, the occurrence of liver fibrosis increased, and it is suitable to adjust the reference range of liver function (26). In short, HUA is a metabolic disease related to habits, customs, age, gender, and inherited factors, and HUA is also correlated with obesity, hypertension, diabetes, renal failure, dyslipidemia, and liver diseases (27–29). Of course, the specific mechanism needs further study.

As for the gut microbiotas, we found that the gut bacterial diversity in HUA group was reduced significantly compared with that of healthy controls and the community of the microbiota was also of significant difference between the two groups. Just as Requena et al. (30) had mentioned the diversity of microbiota was closely associated with our health, the result of our study indicated that the gut microbiota may have altered significantly from a normal healthy status to the development of HUA. In the phyla level, compared with healthy controls, the abundance of Bacteroidetes in HUA group decreased [consistent with the previous research result (31)], while Firmicutes and Actinobacteria increased significantly. At the species level, totally 51 species were of significant difference after removing the low occurrence rate and low abundance species. Among them, 37 species were enriched in the healthy controls and 14 species were enriched in the HUA group. This suggests that maybe it is these significantly different populations in abundance that cause the significant changes in the composition of gut microbiota HUA.

Among the 51 species, compared with the species of healthy controls, the relative abundances of Bacteroidetes (Bacteroides uniformis, caccae, ovatus and plebeius, Alistipes finegoldii, senegalensis, and shahii); Firmicutes (Roseburia hominis, Ruminococcus callidus and lactaris, and Lachnospiraceae bacterium 3_1_46FAA); other (Adlercreutzia equolifaciens, Odoribacter splanchnicus, Butyricimonas synergistica, and Akkermansia muciniphila) decreased in the HUA group; while Firmicutes (Ruminococcus gnabus and torques, Lachnospiraceae bacterium 9_1_43FAA); Actinobacteria (Collinsella aerofaciens) increased. Interestingly, most of the gut microbiomes enriched in HUA group not only had significant positive correlations with the clinical characteristics, but also most of them are responsible for the production of SCFAs, while those microbiomes enriched in the healthy controls had highly negatively correlated with these clinical indexes.

In our study, some species that are known as SCFAs (e.g. butanoate) producer [*Roseburia hominis* (32, 33), *Odoribacter splanchnicus* (34), *Ruminococcus callidus* (35), *Lachnospiraceae bacterium 3_1_46FAA* (36)*, Bacteroides uniformis* (37) and *Butyricimonas synergistica* (38)], together with the metabolic pathway of producing butanoate (CENTFERM-PWY and PWY-6590) were enriched in the healthy controls. Importantly, both of the two pathways had significantly positive correlations with the above bacterials. Especially, the bacterial of *Odoribacter splanchnicus* had the strongest correlation with them (*r* = 0.903 and 0.902 relatively) and had frequent correlations with clinical parameters, *Roseburia hominis* was the bacteria that most negatively correlated with clinical parameters and can produce large amounts of SCFAs, which play key protective roles against inflammation (39–41). As is known to all, *Odoribacter splanchnicus* is expressed in human intestine and its complete genome had been sequenced (42), and now it is recognized as a common butyric-acid producing bacterium, which has been reclassified from Bacteroides splanchnicus (34). In a recent study, *Odoribacter splanchnicus* together with *Akkermansia muciniphila*, has been associated with a healthy fasting serum lipid profile (43). The administration of prebiotics and the use of polyphenols have been stated as effective dietary strategies to modulate gut microbiota composition, which could induce the abundance of friendly bacteria, namely *Akkermansia muciniphila*, associated to health benefits in metabolic syndrome (44–46). Our study evidenced that HUA state may reduce or inhibit the growth of those “beneficial” bacterial members.

Besides, another relevant bacterium was *Adlercreutzia equolifaciens*, which was enriched in the healthy controls. *A. equolifaciens* is involved in metabolizing polyphenols and produced bioactive molecules involved in ameliorating metabolic disorders in obesity and diabetes (47). The study had verified that it can convert ingested isoflavones, which is abundant in legumes and soya beans, into equol (48). Equol has a high affinity for the estrogen receptor (49) and may be a selective estrogen receptor modulator. The incidence of HUA is high in men and postmenopausal women, and studies have concluded that it is the estrogen in premenopausal women that reduces its incidence through the follow ways, such as estrogen can directly affect the kidney excretion of uric acid by regulating transporters (49–51); inhibit the xanthine oxidase system (52); reduce the production of uric acid by maintaining the stability of lipid metabolism (53–55). Thus, we can propose *Adlercreutzia equolifaciens* may make contributions to alleviate the damage of HUA to our body in some estrogen-protection way. This finding is of great significance and maybe can provide a direction for the further exploration of new therapeutic targets for HUA based on gut microbiota.

On the contrary, *Lachnospiraceae bacterium 1_4_56FAA, Ruminococcus gnavuswas and torques* were enriched in the HUA group. Not only did they had positive correlations with most clinical indexes, but also the pathways that are significantly enriched in HUA group, such as superpathway biosynthesis of 5-aminoimidazole ribonucleotide (PWY-6122, PWY-6277 and PWY-6121), aromatic amino acids and chorismate synthesis (COMPLETE-ARO-PWY, ARO-PWY and PWY-6163), and L-lysine, L-threonine and L-methionine biosynthesis (PWY-724). Consistent with our results, all of the current studies have shown that the above three bacterials are closely related to immune inflammation in different diseases (56–60), though the inflammation indicators (WBC, PLT, NEUT, and NLR) were slightly, but not significantly, higher in the HUA group than in the healthy controls. Especially, *R. gnavus* can produce specific antigens and stimulates immune cells to produce corresponding antibodies, thus increasing inflammation (61, 62). As to the functional alterations, 5-aminoimidazole ribonucleotide is the key intermediate for purine nucleotide biosynthesis, the increased biosynthesis of 5-aminoimidazole ribonucleotide may increase the production of purines, thereby increase the level of uric acid. Besides, the aromatic amino acids and chorismate can act as substrates in other secondary metabolite pathways, such as indole derivatives and phenolic compounds, which are just the metabolites of our gut microbiotas. So far, no studies have been able to confirm the correlations between these structural and functional alterations of gut microbiota in HUA, but their significant alterations suggest that they may have important physiological significance in the occurrence and development of HUA, which is a research direction worthy for our further study.

There are some limitations in our study. First, this is a cross-sectional study, and it is not able to verify the causality between the discovered species and HUA, hence, further germ-free mice studies are still needed. Second, the study subjects come from the same region, thus, it is better to conduct a multicenter study from different regions owing to the influence of different regions and eating habits on the gut microbiota. Third, the gender in the HUA group is male adult, therefore, it is necessary to be cautious temporarily to apply the results obtained in this article to the female, minors or other population.

In conclusion, our study demonstrated the structural and functional alterations of gut microbiota in males with HUA and high levels of liver enzymes. In our study, the main clues of the correlation mechanisms between HUA and gut microbiome point to the pathways that can produce 5-aminoimidazole ribonucleotide, aromatic amino acids, and chorismate, which were enriched in the HUA group; the pathways that can produce SCFAs and the gut microbiotas that can produce SCFAs and equol were enriched in healthy controls. Generally, the potential mechanisms underlying the gut microbiome-Equol/SCFAs-uric acid accumulation link remains much for further exploration. The novel correlations between some microbiota species/pathways and uric acid accumulation could provide brand new directions for specific microbiota-targeted therapies.

## Data Availability Statement

The datasets presented in this study can be found in online repositories. The names of the repository/repositories and accession number(s) can be found at: https://www.ebi.ac.uk/ena,PRJEB48022.

## Ethics Statement

The studies involving human participants were reviewed and approved by Ethics Committee from the First Affiliated Hospital of Zhengzhou University. The patients/participants provided their written informed consent to participate in this study.

## Author Contributions

SD and SS: Conceptualization. JC, YW, and AL: Methodology and formal analysis. QQ, YZ, WL, TL, and XG: Resources. SS: Writing and original draft preparation. SD, LT, and AL: Writing and review and editing. JC and SY: Visualization. All authors have read and agreed to submit the manuscript.

## Funding

This research was equally supported and funded by the Henan Province Medical Science and Technology Research Plan (LHGJ20200311 and LHGJ20200279), Chinese National Science and Technology Major Project (2018ZX10305410), Henan Province Youth Talent Promotion Project (Grant No.2021HYTP052), the Henan Province Postdoctoral Research Grant (001801005).

## Conflict of Interest

The authors declare that the research was conducted in the absence of any commercial or financial relationships that could be construed as a potential conflict of interest.

## Publisher's Note

All claims expressed in this article are solely those of the authors and do not necessarily represent those of their affiliated organizations, or those of the publisher, the editors and the reviewers. Any product that may be evaluated in this article, or claim that may be made by its manufacturer, is not guaranteed or endorsed by the publisher.

## Supplementary Material

Supplementary Table The detailed experimental results between HUA group and healthy controls.

## Abbreviations

WC: waist circumference
BMI: body mass index
FPG: fasting plasma glucose
DBP: diastolic blood pressure
SBP: systolic blood pressure
AST: aspartate aminotransferase
ALT: alanine aminotransferase
GGT: glutamyl transpeptidase
TBIL: total bilirubin
IBIL: indirect bilirubin
SUA: serum uric acid
Scr: serum creatinine
TC: total cholesterol
TG: triglyceride
LDL: low-density lipoprotein
HDL: high-density lipoprotein
WBC: white blood cell
PLT: platelet
NEUT: absolute value of neutrophil
NLR: neutrophil to lymphocyte ratio
SCFAs: short-chain fatty acids.
